# Diagnostic delay as a prognostic factor for non-response to treatment and relapse in Mexican pediatric patients with central nervous system tumors

**DOI:** 10.3389/fped.2026.1900510

**Published:** 2026-10-20

**Authors:** Juan-Carlos Pérez-De Marcos, Manuel Castillejos-López, Arnoldo Aquino-Gálvez, Liliana Velasco-Hidalgo, Rocío Cárdenas-Cardos, Ana María Niembro-Zúñiga, Armando De Uña-Flores, Marta Zapata-Tarrés, Luz-María Torres-Espíndola

**Affiliations:** 1 Laboratorio de Farmacología, Instituto Nacional de Pediatría, Ciudad de México, México; 2 Servicio de Oncología, Instituto Nacional de Pediatría, Ciudad de México, México; 3 Laboratorio de Investigación en Epidemiología e Infectología, Instituto Nacional de Enfermedades Respiratorias Ismael Cosío Villegas, Ciudad de México, México; 4 Laboratorio de Biología Molecular, Instituto Nacional de Enfermedades Respiratorias “Ismael Cosío Villegas”, Ciudad de México, México; 5 Servicio de Radiología e Imagen, Instituto Nacional de Pediatría, Ciudad de México, México; 6 Titular de la Comisión Coordinadora de Institutos Nacionales de Salud y Hospitales de Alta Especialidad, Ciudad de México, México

**Keywords:** central nervous system tumors, diagnostic delay, Mexican pediatric, non-response, relapse

## Abstract

**Background:**

Central nervous system tumors (CNST) are among the most common solid tumors in children and represent a major cause of cancer-related morbidity and mortality. Early diagnosis is essential to improve treatment outcomes; however, nonspecific symptoms often lead to diagnostic delays. Prolonged time to diagnosis may allow disease progression and negatively affect treatment response. Understanding the impact of diagnostic delays can help identify barriers to timely care and improve clinical outcomes in pediatric patients.

**Objective:**

This study aimed to explore the link between diagnostic delays and relapse on treatment non-response in pediatric patients with central nervous system tumors (CNST).

**Methods:**

A cohort of 111 patients with newly diagnosed CNST tumors was evaluated. Treatment response was assessed using Response Assessment in Neuro-Oncology (RANO) criteria. A diagnostic delay was considered when the diagnosis was made more than 1, 3, or 6 months after the initial clinical presentation. Kaplan–Meier curves were plotted to assess the probability of treatment response by diagnostic delay of more than 1, 3, and 6 months.

**Results:**

The median time from symptom onset to diagnosis was 80 days (Q_25_29–Q_75_195). Kaplan–Meier analysis showed that diagnostic delays >1 and >3 months were associated with a higher probability of non-response to treatment (*p* = 0.014 and *p* = 0.004, respectively). In multivariable Cox analysis adjusted for age and sex, delays >1 month (HR 2.003, 95% CI 1.23–3.24; *p* = 0.005) and >3 months (HR 1.86, 95% CI 1.20–2.88; *p* = 0.005) were associated with non-response. Diagnostic delays >1 month (HR 2.690, 95% CI 1.19–6.34; *p* = 0.043) and >3 months (HR 2.083, 95% CI 1.08–3.96; *p* = 0.048) were also associated with relapse.

**Conclusion:**

A delay in diagnosis of more than 1 and 3 months was significantly associated with lack of response to treatment and relapse in patients diagnosed with CNST.

## Introduction

Central nervous system tumors (CNST) in children are the second most common cause of childhood cancer after leukemia (1).

While the overall 5-year survival rate in some high-income countries ranges from 80% to 90%, it varies between 5% and 60% in low- and middle-income countries. Consequently, data from Mexico indicate a 50% long-term survival rate (2, 3).

The effectiveness of cancer therapy has improved significantly in recent decades. However, childhood cancer can often be fatal without timely diagnosis and appropriate treatment. Unlike adult cancer, there is currently no evidence-based population-based screening programs or effective lifestyle risk reduction strategies to improve outcomes in children (4, 5). Delayed cancer diagnoses and high instances of advanced disease are linked to a higher risk of infant mortality, highlighting the importance of timely detection and proactive health measures (6, 7).

Timely diagnosis is essential for the successful treatment of childhood cancer, as in some cases it can prevent disease progression and improve patient prognosis. However, CNST in children are often initially misdiagnosed as other more common pediatric conditions, such as migraines, gastroenteritis, or psychological or behavioral problems, resulting in delays in diagnosis (8). It has been reported that some of the main factors related to diagnostic delay are: age at diagnosis, parental education level, cancer type, tumor location, and symptom presentation (9). The literature on the impact of delayed diagnosis of CNST in pediatrics is limited. Most existing studies focus on the factors that contribute to diagnostic delays without examining their effects on patient response. This article aims to explore the relationship between diagnostic delays and relapse on treatment response in pediatric patients with CNST**,** highlighting its importance for advancing pediatric diagnostics.

## Methodology

### Study population

Electronic records were reviewed for clinical and epidemiological data, as well as treatment response outcomes, of pediatric patients with a histopathological diagnosis of CNST according to the latest WHO 2016 classification, from October 2018 to October 2021. All patients included in this study were part of a study approved by institutional committees with registration number 061/2018.

### Treatment response assessment

The Response Assessment in Neuro-Oncology (RANO) criteria (10) are standardized guidelines used to evaluate how brain tumors respond to therapy. These criteria should be applied at diagnosis, three months after chemoradiotherapy (when applicable, to reduce the risk of false-positive MRI results), at predetermined time points according to the treatment protocol (to assess partial response and guide decisions about continuing or modifying therapy), and at the end of treatment to initiate post-treatment surveillance. However, in this cohort, the final assessment of treatment response was conducted at the end of the treatment protocol, based on an initial imaging evaluation at the time of diagnosis.

Complete response (CR) is defined as the complete disappearance of all measurable and non-measurable lesions sustained for at least 4 weeks, with no new lesions; partial response (PR) is defined as a ≥ 50% decrease in the sum of the perpendicular diameters of measurable lesions compared to the baseline study for at least 4 weeks; stable disease (SD): criteria for complete response, partial response, or progression of disease (PD) are not met; disease progression: a ≥ 25% increase in the sum of the perpendicular diameters compared to the minimum measurement obtained or compared to the baseline study.

Patients with CR and PR were classified as responders, and those with SD and PD were classified as non-responders. This evaluation was conducted in collaboration with a specialist physician assigned to the Radiology and Imaging Department and a Pediatric Oncologist.

### Diagnostic delay (DD)

Diagnostic delay refers to the period of time between the onset of symptoms and the establishment of the CNST diagnosis. The exact date of diagnosis was defined as the date of the definitive event that confirmed the disease and authorized the initiation of oncological treatment. The analysis was performed using independent cutoffs at >1, >3, and >6 months.

### Statistical analysis

Poor response rates to treatment were estimated using the Kaplan–Meier method, allowing for time-to-event analysis. To assess differences in outcomes between patient groups, comparisons were performed using the log-rank test.

### Multivariate cox regression analysis

Hazard ratios were estimated using Cox proportional hazards regression. A *p*-value < 0.05 was considered statistically significant. The model was adjusted for sex and age to assess whether diagnostic delay was associated with non-response to treatment. All statistical analyses were performed using SPSS version 21.0 (IBM Corp., Armonk, NY, USA).

## Results

### Clinical-epidemiological profile of the cohort

One hundred eleven patients with a primary tumor of the central nervous system were analyzed. In terms of diagnostic confirmation, 104 patients (94%) were diagnosed via histopathology (surgical resection or biopsy), while 7 patients (6%) with secreting germ cell tumors were diagnosed via CSF/serum tumor markers (CSF beta-hCG and/or alpha-fetoprotein) and neuroimaging. Regarding primary therapy, 95 patients (85.6%) underwent surgical resection (complete or partial) as their initial therapeutic step prior to adjuvant therapy, whereas 16 patients (14.4%) underwent biopsy only followed by immediate chemotherapy or radiotherapy. The predominant sex was male, *N* = 61 (55%); the median age was 12 years; high-grade tumors were the most frequent; and disease progression predominated as the treatment response. Table 1.

**Table 1:** Characteristics of the study population.

Characteristic	N (%)
Sex	
Men	61 (55)
Women	50 (45)
Age at diagnosis (years)	
Median	12
Malignancy grade	
High grade	71 (64)
Low grade	40 (36)
Time to diagnosis (months)	
Delay in diagnosis (>1)	80 (72.1)
Delay in diagnosis (>3)	52 (46.8)
Dalay in diagnosis (>6)	32 (28.8)

In the analyzed cohort, the majority of central nervous system tumors (CNST) were of glioma origin, accounting for 61 cases (55%). This was followed by embryonal tumors with 39 cases (35%), germ cell tumors with 7 cases (6%), and other types of tumors, which consisted of 4 cases (4%), as shown in Figure 1.

**Figure 1:**
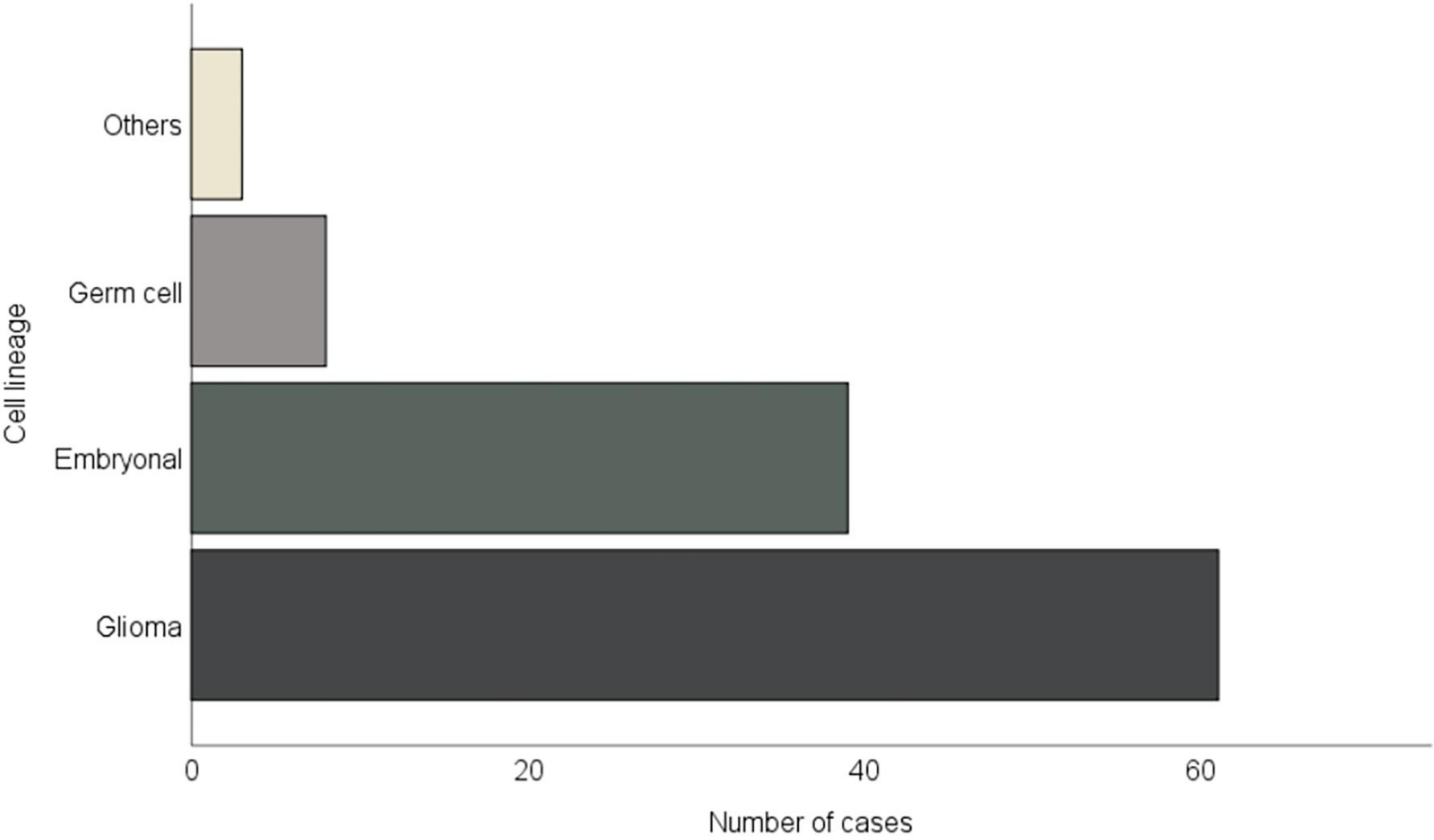
Frequency of tumors according to cell lineage.

Regarding treatment responses, a complete response was observed in 22 patients (19.8%), a partial response in 14 patients (12.6%), stable disease in 24 patients (21.6%), and disease progression in 51 patients (46%), as illustrated in Figure 2.

**Figure 2:**
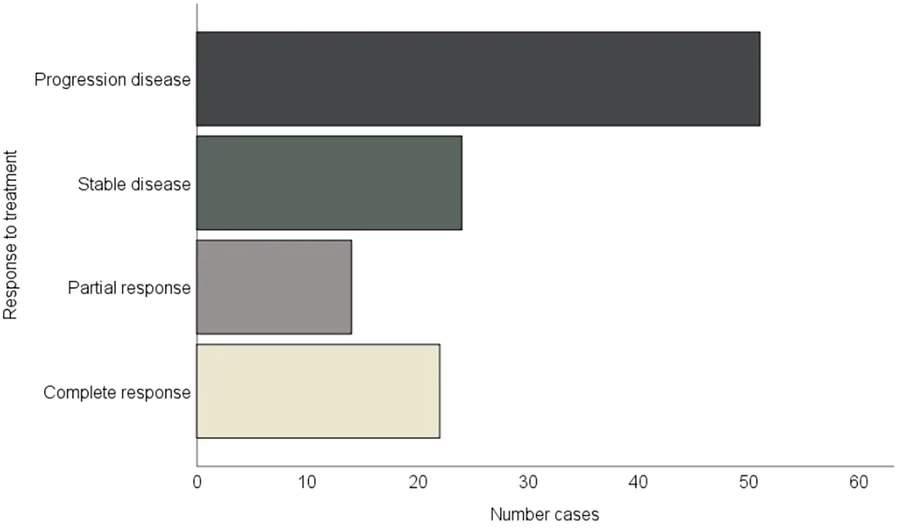
Response to treatment in the cohort.

The delay in diagnosis for each patient was recorded from the date of symptom onset to the date of CNST diagnosis. It was found that 80 patients (72.1%) were diagnosed after more than 30 days (>1 month). A second group of 52 patients (46.8%) was diagnosed after more than 90 days (>3 months); 32 (28.8%) were diagnosed after more than 180 days (>6 months) Figure 3.

**Figure 3:**
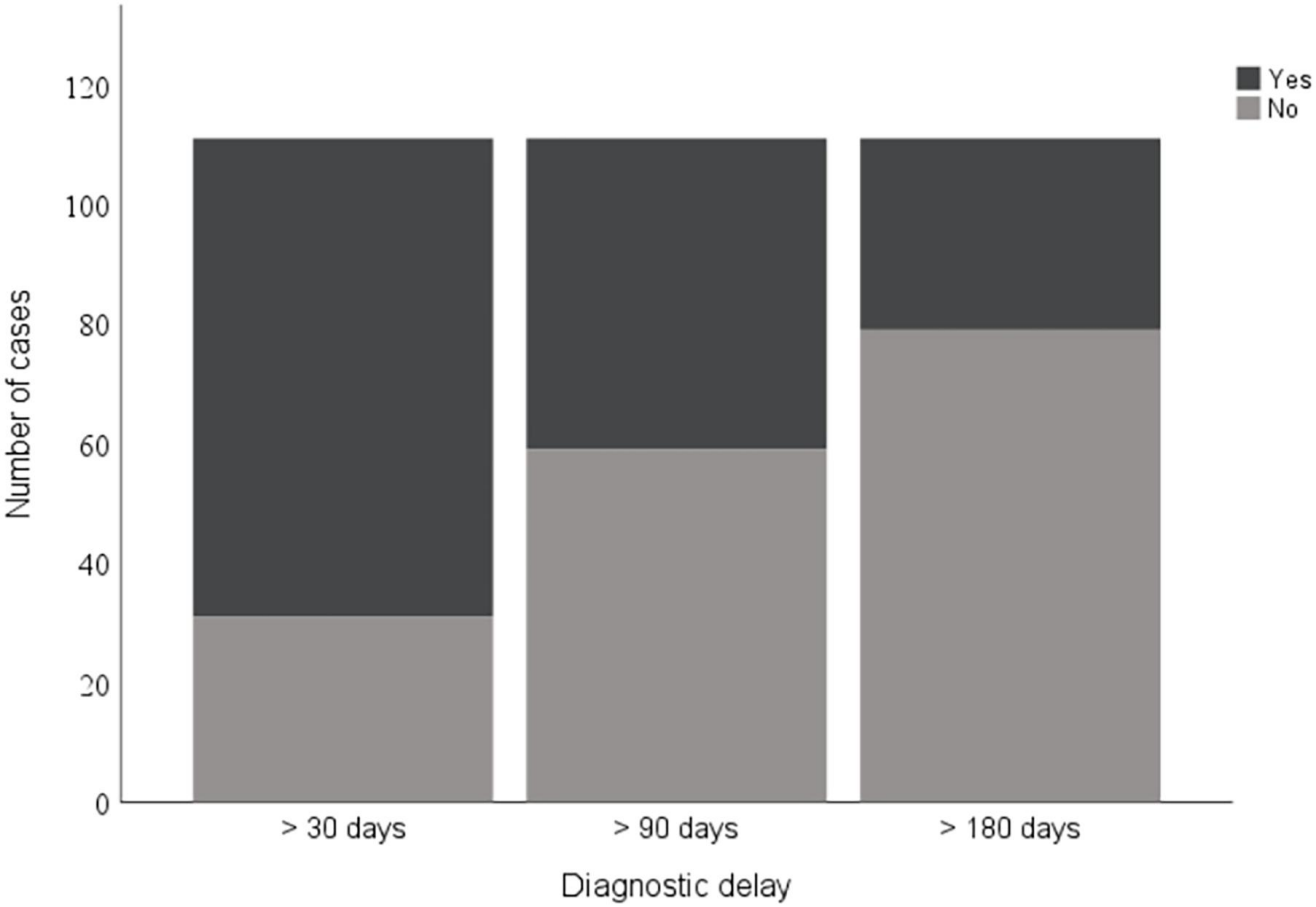
Number of cases according to the delay in diagnosis.

### Probability curves for response to treatment as a function of a delay of more than 1 month, more than 3 months, and more than 6 months from diagnosis

Kaplan–Meier curves were generated to evaluate the probability of treatment response, comparing diagnostic delays of more than 1, 3, or 6 months after the initial clinical presentation. The analysis revealed that a diagnostic delay of one month or longer was significantly associated with an increased probability of response to treatment (*p* = 0.014). Similarly, delays of three months or more showed a stronger association (*p* = 0.004), whereas delays of six months or more did not reach statistical significance (*p* = 0.111) (Figures 4A–C). Kaplan–Meier curves were also constructed for relapse; however, no statistically significant associations were detected.

**Figure 4:**
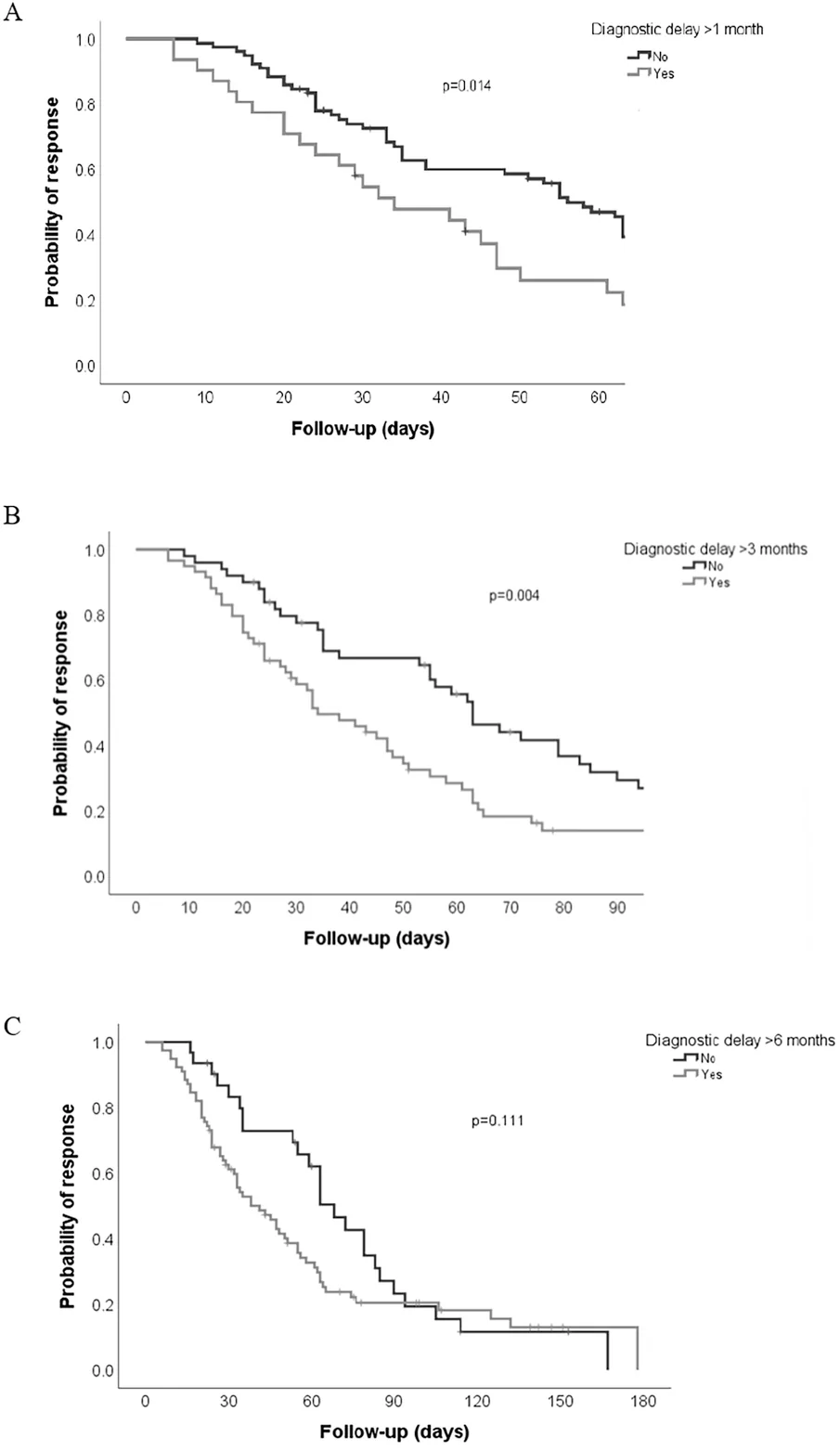
Probability of response to treatment according to the delay of > 1 **(A)**, > 3 **(B)**, and >6 months **(C)** in the diagnosis.

### Effect of diagnostic delay on non-response and relapse

A multivariate analysis was performed to estimate the influence of time to diagnosis on non-response to treatment and relapse, adjusted for sex and age. These results are shown in Table 2. A significant association was found between a diagnostic delay of >1 month and >3 months and non-response to treatment in patients with CNST: [HR 2.003 (95% CI 1.23-3.24), *p* = 0.005] and [HR 1.86 (95% CI 1.20–2.88), *p* = 0.005], respectively. Similarly, we found a significant association between diagnostic delay of >1 month or >3 months and relapse: [HR 2.690 (95% CI 1.19–6.34), *p* = 0.043] and [HR 2.083 (95% CI 1.08–3.96), *p* = 0.048] Table 3.

**Table 2:** Cox proportional hazards models evaluating diagnostic delay as a predictor of non-response, using diagnostic delay cutoffs: model 1 (>1 month) and model 2 (>3 months).

Variables	Model 1: Diagnostic delay >1 month HR (95% CI)	*p*-value	Model 2: Diagnostic delay >3 months HR (95% CI)	*p*-value
Diagnostic delay	2.003 (1.23–3.24)	0.005*	1.86 (1.20–2.88)	0.005*
Age (years)	1.32 (0.79–2.21)	0.28	0.93 (0.89–0.98)	0.006
Male sex	1.71 (1.10–2.64)	0.015	1.38 (0.87–2.20)	0.16

HR: hazard ratio. CI: confidence interval. * Statistical significance was calculated using a multivariate Cox regression.

**Table 3:** Cox proportional hazards models evaluating diagnostic delay as a predictor of relapse, using diagnostic delay cutoffs: model 1 (>1 month) and model 2 (>3 months).

Variable	Model 1: Diagnostic delay >1 month HR (95% CI)	*p*-value	Model 2: Diagnostic delay >3 months HR (95% CI)	*p*-value
Diagnostic delay	2.690 (1.19–6.34)	0.043*	2.083 (1.08–3.96)	0.048*
Age (years)	0.958 (0.883–1.040)	0.28	0.963 (0.88–1.05)	0.418
Male sex	0.997 (0.41–2.41)	0.894	1.18 (0.49–2.78)	0.71

HR: hazard ratio. CI: confidence interval. * Statistical significance was calculated using a multivariate Cox regression.

.

## Discussion

This study is the first to demonstrate the effects of delayed diagnosis on treatment response in a cohort of Mexican pediatric patients with CNST.

Research on diagnostic delays and response rates in children with CNST has produced inconsistent findings to date. A meta-analysis conducted by Cotache et al. in 2023 identified several key factors that contribute to delays in cancer diagnosis. These factors include geographical, social, financial, health system, and health policy barriers, which restrict access to cancer care in low- and middle-income countries. However, the meta-analysis did not assess the impact of these delays on patients (11).

The results to date are scarce and contradictory for the pediatric population. Ocak S et al. (2021) found that delays in diagnosis did not affect survival in pediatric patients with solid tumors and lymphomas (12).

Barragán E et al. (2020) found that high-grade brain tumors had a higher risk of dying when the global delay interval (GDI) was between 3 and 6 months compared to < 3 months (7). There is limited literature linking diagnostic delays to overall survival rates in pediatric patients. Timely diagnosis and prompt treatment initiation depend on early referral to tertiary care and effective tumor management. Diagnosing cancer as early as possible is a crucial public health strategy because it increases the likelihood of successful treatment. When treatment is delayed or unaffordable, the chances of survival decrease, treatments become more complicated and lengthier, and overall healthcare costs rise.

In certain tumor cases, a delayed diagnosis may merely extend the duration of the disease without resulting in permanent consequences. However, in other instances, the absence of a timely diagnosis and subsequent treatment can lead to significant adverse effects. For example, a delay in diagnosing a tumor can result in disease progression and ultimately result in mortality.

These findings suggest that the relationship between diagnostic delay and treatment response may not be linear and may vary according to tumor biology and aggressiveness. While shorter diagnostic delays were associated with poorer treatment responses and increased disease progression in our cohort, delays of 6 months or more did not show a statistically significant association with treatment non-response. This apparent discrepancy may be explained, at least in part, by the “waiting-time paradox” and length-bias artifact, whereby patients with more aggressive tumors tend to present earlier because of rapidly progressive symptoms. In contrast, patients with less aggressive tumors may experience longer prediagnostic intervals.

Interestingly, our study showed that diagnostic delays of 6 months or more did not reach statistical significance regarding treatment non-response. This finding highlights a common epidemiological phenomenon in delay studies known as the ‘waiting time paradox.’ This paradox occurs because patients with highly aggressive or high-grade tumors present with rapid, severe clinical symptoms that prompt urgent medical evaluation, resulting in shorter diagnostic intervals but inherently worse biological outcomes. Conversely, patients with low-grade or slow-growing tumors survive long enough to experience extended pre-diagnostic delays (>6 months) without showing immediate treatment failure. This length-bias artifact underscores that biological tumor aggressiveness remains a key confounder when analyzing extremely long diagnostic delays.

Early diagnosis of pediatric tumors, such as ependymomas, presents a challenge for healthcare providers. This is primarily due to the nonspecific clinical manifestations of brain tumors, which can resemble symptoms of other, more common conditions. Additionally, the age at which symptoms are recognized can complicate the situation (13).

Research has indicated that several factors can influence the duration of the prediagnostic interval, including the tumor's histology and location (7, 14, 15). Other well-studied factors that can lead to delayed diagnosis include caregiver characteristics, parents' educational levels, prior knowledge of the disease, and cultural beliefs (16, 17).

Response to treatment was the primary endpoint of this study, but how a radiographic response relates to overall survival (OS) is not the same across pediatric CNS tumor types. The RANO criteria were developed to standardize response assessment in children and combine tumor measurements with clinical status and leptomeningeal findings; they remain bidimensional measurements, however, and have not been validated against survival (18). In entities where objective responses occur, they have tracked with survival: response-adapted radiotherapy in intracranial germinoma, for example, produced a 5-year OS of 98% in localized disease (19). In diffuse intrinsic pontine glioma, the factors that predict longer OS are clinical and radiological characteristics at diagnosis rather than the response measured during treatment (20). Relapsed medulloblastoma behaves differently: objective responses in early-phase trials are infrequent, and response by itself has been an imperfect predictor of survival (21). Pediatric low-grade glioma has an excellent OS above 90%; event-free survival carries more weight there, and pseudo-progression after radiotherapy can confound early response assessment (22). Our study was not designed or powered to detect differences in OS, and survival follow-up is still ongoing. The response rates reported here should therefore be read as an early sign of anti-tumor activity, not as a predictor of OS.

### Limitations

This study has limitations that should be taken into account when interpreting our findings. First, diagnostic delay was defined using specific cut-off points for the time elapsed between symptom onset and the exact date of diagnosis. However, determining the exact date of symptom onset retrospectively from electronic records is subjective and susceptible to recall bias. We largely based symptom onset on clinical notes and caregiver reports, which may be incomplete. This can potentially lead to an underestimation or misclassification of the true interval. Such misclassification could have resulted in some patients being incorrectly categorized across the 1-, 3-, and 6-month cutoffs, thereby affecting the observed associations with non-response to treatment and relapse. Second, we classified treatment response according to the RANO criteria (complete response, partial response, stable disease, and progressive disease). We assessed response to treatment six weeks after radiotherapy for all tumors, six weeks after surgical resection, and after every 3–4 cycles of chemotherapy, depending on the tumor; however, for this study, we assessed response at the end of the entire course of treatment. Consequently, some patients may have been misclassified, and the association between diagnostic delay and non-response should be interpreted with caution. To validate these results, future prospective studies should use standardized, systematic methods to collect data on symptom onset dates.

## Conclusion

Pediatric patients with CNS tumors who had a diagnostic delay of >1 month or >3 months were associated with poor treatment responses and a higher risk of disease progression. Given the high rates of morbidity and mortality associated with CNST, it is crucial to emphasize the early diagnosis of this type of tumor among various childhood cancers, starting at the primary care level. This approach aims to reduce the time to diagnosis and ensure timely referrals to hospitals that can provide comprehensive treatment. As a result, patients cannot only experience prolonged survival but also an improved quality of life, despite the complications that may arise from different treatment modalities or from the progression of the disease.

Addressing the various risk factors identified in the literature that contribute to delayed diagnosis, along with implementing targeted training programs for primary care physicians to help them recognize the early symptoms of these tumors, would help reduce the delay in diagnosing central nervous system tumors (CNST).

## Data Availability

The raw data supporting the conclusions of this article will be made available by the authors, without undue reservation.

## References

[1] Correa-Correa V Núñez-Enríquez JC Mezei G Rivera-Luna R Peñaloza-González JG Rivas-Carrillo SD. Extremely low-frequency magnetic fields (ELF-MF) and radiofrequency: risk of childhood CNS tumors in a city with elevated ELF-MF exposure. Environ Res. (2025) 286(Pt 2):122858. 10.1016/j.envres.2025.122858 40957475 PMC12799243

[2] Rodriguez-Romo L Olaya Vargas A Gupta S Shalkow-Klincovstein J Vega-Vega L Reyes-Lopez A. Delivery of pediatric cancer care in Mexico: a national survey. J. Glob. Oncol. (2018) 4:1–12. 10.1200/JGO.17.00238 30084750 PMC6223522

[3] Rivera-Luna R Zapata-Tarres M Shalkow-Klincovstein J Velasco-Hidalgo L Olaya-Vargas A Finkelstein-Mizrahi N. The burden of childhood cancer in Mexico: implications for low-and middle-income countries. Pediatr Blood Cancer. (2017) 64(6):e26366. 10.1002/pbc.26366 27905680

[4] Mullen CJR Barr RD Franco EL. Timeliness of diagnosis and treatment: the challenge of childhood cancers. Br J Cancer. (2021) 125:1178. 10.1038/s41416-021-01548-x 34471259 PMC8651632

[5] Bohaligah KM Bohaligah MM Bohaligah SM. Preventive strategies for pediatric health in primary healthcare: a systematic review. Cureus. (2025) 17:e78719. 10.7759/cureus.78719 40065858 PMC11891502

[6] Vásquez L Montoya J Ugaz C Ríos L León E Maza I. ONCOPEDS: a mobile application to improve early diagnosis and timely referral in childhood cancer in a low-and middle-income country—a pilot study. Pediatr Blood Cancer. (2021) 68(4):e28908. 10.1002/pbc.28908 33501779

[7] Barragán-Pérez EJ Altamirano-Vergara CE Alvarez-Amado DE García-Beristain JC Chico-Ponce-de-León F González-Carranza V. The role of time as a prognostic factor in pediatric brain tumors: a multivariate survival analysis. Pathol Oncol Res. (2020) 26(4):2693–701. 10.1007/s12253-020-00875-3 32661835

[8] Goldman RD Cheng S Cochrane DD. Improving diagnosis of pediatric central nervous system tumours: aiming for early detection. CMAJ. (2017) 189(12):E459–63. 10.1503/cmaj.160074 27895146 PMC5367991

[9] Pedersen LH Wahlberg A Cordt M Schmiegelow K Dalton SO Larsen HB. Parent’s perspectives of the pathway to diagnosis of childhood cancer: a matter of diagnostic triage. BMC Health Serv Res. (2020) 20:969. 10.1186/s12913-020-05821-2 33092610 PMC7584100

[10] Chukwueke UN Wen PY. Use of the response assessment in neuro-oncology (RANO) criteria in clinical trials and clinical practice. CNS Oncol. (2019) 8:CNS28. 10.2217/cns-2018-0007 30806082 PMC6499019

[11] Cotache-Condor C Kantety V Grimm A Williamson J Landrum KR Schroeder K. Determinants of delayed childhood cancer care in low- and middle-income countries: a systematic review. Pediatr Blood Cancer. (2023) 70:e30175. 10.1002/pbc.30175 36579761 PMC10966942

[12] Ocak S Sen HS Turkkan E. The time to diagnosis and survival in children with solid tumors and lymphoma: results from a single center in Turkey. Pediatr Hematol Oncol. (2022) 39(2):121–31. 10.1080/08880018.2021.1951903 34281453

[13] Perez-Roca E Negreiros T Casavilca-Zambrano S Ojeda-Medina L Díaz-Coronado R. Prognostic factors of pediatric ependymomas at a national cancer reference center in Peru. Front Oncol. (2024) 13:1331790. 10.3389/fonc.2023.1331790 38298447 PMC10828566

[14] Shanmugavadivel D Liu JF Murphy L Wilne S Walker D. Accelerating diagnosis for childhood brain tumours: an analysis of the HeadSmart UK population data. Arch Dis Child. (2020) 105(4):355–62. 10.1136/archdischild-2018-315962 31653616

[15] Coven SL Stanek JR Hollingsworth E Finlay JL. Delays in diagnosis for children with newly diagnosed central nervous system tumors. Neurooncol Pract. (2018) 5(4):227–33. 10.1093/nop/npy002 31386013 PMC6655489

[16] Lu P Raynald JR Liu W Gong J Sun T Li C. Factors impacting time to diagnosis in pediatric CNS tumors in Chinese children. Support Care Cancer. (2021) 29:3633–42. 10.1007/s00520-020-05863-6 33179135

[17] Faruqui N Joshi R Martiniuk A Lowe J Arora R Anis H. A health care labyrinth: perspectives of caregivers on the journey to accessing timely cancer diagnosis and treatment for children in India. BMC Public Health. (2019) 19:1613. 10.1186/s12889-019-7911-x 31791308 PMC6889559

[18] Erker C Tamrazi B Poussaint TY Mueller S Mata-Mbemba D Franceschi E. Response assessment in paediatric high-grade glioma: recommendations from the response assessment in pediatric neuro-oncology (RAPNO) working group. Lancet Oncol. (2020) 21:e345–56. 10.1016/S1470-2045(20)30379-X 32502458

[19] Chukwueke UN Wen PY. Use of the Response Assessment in Neuro-Oncology (RANO) criteria in clinical trials and clinical practice. CNS Oncol. (2019) 8: CNS28. 10.2217/cns-2018-0007 30806082 PMC6499019

[20] Jansen MH Veldhuijzen van Zanten SE Sanchez Aliaga E Heymans MW Warmuth-Metz M Hargrave D. Survival prediction model of children with diffuse intrinsic pontine glioma based on clinical and radiological criteria. Neuro Oncol. (2015) 17:160–6. 10.1093/neuonc/nou104 24903904 PMC4483042

[21] Bautista F Fioravantti V De Rojas T Carceller F Madero L Lassaletta A. Medulloblastoma in children and adolescents: a systematic review of contemporary phase I and II clinical trials and biology update. Cancer Med. (2017) 6:2469–78. 10.1002/cam4.1171 PMC5673921 28980418

[22] Ludmir EB Mahajan A Paulino AC Jones JY Ketonen LM Su JM. Increased risk of pseudoprogression among pediatric low-grade glioma patients treated with proton versus photon radiotherapy. Neuro Oncol. (2019) 21:686–95. 10.1093/neuonc/noz042 30753704 PMC6502497

